# Aspects of the Tumor Microenvironment Involved in Immune Resistance and Drug Resistance

**DOI:** 10.3389/fimmu.2021.656364

**Published:** 2021-05-27

**Authors:** Khalil Khalaf, Doris Hana, Jadzia Tin-Tsen Chou, Chandpreet Singh, Andrzej Mackiewicz, Mariusz Kaczmarek

**Affiliations:** ^1^ Department of Cancer Diagnostics and Immunology, Greater Poland Cancer Center, Poznań, Poland; ^2^ Department of Cancer Immunology, Poznan University of Medical Sciences, Poznań, Poland

**Keywords:** TME (tumor microenvironment), HIF - hypoxia inducible factor, CAF, microRNA (miR), MDSC (myeloid-derived suppressor cells), tumor associated macrophage (TAM), Treg - regulatory T cell, TGF - β1

## Abstract

The tumor microenvironment (TME) is a complex and ever-changing “rogue organ” composed of its own blood supply, lymphatic and nervous systems, stroma, immune cells and extracellular matrix (ECM). These complex components, utilizing both benign and malignant cells, nurture the harsh, immunosuppressive and nutrient-deficient environment necessary for tumor cell growth, proliferation and phenotypic flexibility and variation. An important aspect of the TME is cellular crosstalk and cell-to-ECM communication. This interaction induces the release of soluble factors responsible for immune evasion and ECM remodeling, which further contribute to therapy resistance. Other aspects are the presence of exosomes contributed by both malignant and benign cells, circulating deregulated microRNAs and TME-specific metabolic patterns which further potentiate the progression and/or resistance to therapy. In addition to biochemical signaling, specific TME characteristics such as the hypoxic environment, metabolic derangements, and abnormal mechanical forces have been implicated in the development of treatment resistance. In this review, we will provide an overview of tumor microenvironmental composition, structure, and features that influence immune suppression and contribute to treatment resistance.

## Introduction

Experimental observations of tumorigenesis show that tumor cells transition from being transformed and benign to an invasive malignant state. This process is the result of genome instability, in which cells lose their ability to fully differentiate and mature, resulting in the loss of contact inhibition (1).

Various studies have shown that a large majority of cancer-related deaths were attributed to distant metastasis (2, 3). Stephen Paget was the first to hypothesize on what he described as his “seed and soil” theory. In this hypothesis, tumor cells with metastatic potential (i.e. the seed) were inclined to migrate towards specific sites that nurtured and enhanced growth sites (i.e. the soil). This is the earliest publication hypothesizing the importance of the “tumor microenvironment” (TME) in the development of metastases (4). It is known from extensive literature that the metastatic cascade starts with tumor cell dissociation from the cancer niche, followed by extravasation into capillary and lymphatic systems and along nerves, all the while evading immune surveillance. This process culminates in the invasion of distant sites (5). However, metastatic potential develops long before the tumor is ever detected. In the initial stages of primary tumor formation, the accumulation of both genetic and genomic instabilities lead to the development of phenotypic variants with metastatic capacity (6). Furthermore, these variants have the ability to resist apoptosis and circumvent immune defenses by using various soluble factors that are released by malignant and non-malignant tumor-supporting cells (7). These variants in combination with said soluble factors constitute what is now known as the TME.

Additionally, the TME induces chemotherapeutic resistance through acquired or *de novo* mechanisms. In acquired multi-drug resistance (MDR), the expression of ATP-binding cassettes (ABCs), oncogene activation, and tumor-suppressor gene deregulation are achieved *via* cellular crosstalk and cell-to-TME-matrix interaction. Previously-exposed cancer cells acquire phenotypic changes that lead to resistance to subsequent therapy (8). On the other hand, in *de novo* resistance, it has been shown that after exposure to therapy, stromal tissue within the TME provides refuge to a subpopulation of cancer cells and renders them chemo-resistant by inducing stemness (9).

The vast arsenal that is weaponized by the TME in the course of neoplastic disease is currently the topic of great research interest, and the available literature is daunting for researchers and practicing oncologists alike. This review aims to present an overview of the cells and structure of the TME, and its unique characteristics that induce drug resistance and metastasis that remain significant challenges in the treatment of cancer.

## The Tumor Microenvironment and Its Role in Immune Surveillance

Stem cells (SCs) are unspecified cells with the ability to differentiate into multiple cell types to maintain tissue homeostasis. They reside in a specific microenvironment called a stem cell niche, which consists of and is sustained by different soluble factors (10). Tissue homeostasis is balanced and maintained in a way that prevents SC depletion and overactive proliferation. This is achieved by choosing alternate fates: the SC is selected for senescence (i.e. death), or self-renewal (i.e. proliferation) through interactions with other cells and molecular signals within the microenvironment (11). Just like the stem cell niche of healthy tissues, the tumor microenvironment (TME) is very heterogeneous and is a complex component of solid tumors. The TME comprises a diverse cellular and acellular milieu in which cancer stem cells (CSCs) develop and thrive, and various stromal and immune cells are recruited to form and maintain this self-sustained environment (12). Stromal and tumor cell crosstalk has been recognized as crucial for the promotion of a well-organized TME, leading to effective immune evasion, ECM remodeling, and angiogenesis (7).

## Cells and Components of the TME Involved in Suppression of the Anti-Tumor Response

### Stromal Cells

#### Vascular and Lymphatic Endothelial Cells

Neo-angiogenesis is promoted by both tumor and endothelial cells (ECs). Both vascular and lymphatic systems are implicated in early metastasis, with soluble VEGFA promoting vascular EC proliferation, while VEGFC, VEGFD, and VEGFR-3 promoting lymphatic EC proliferation (13, 14).

Tumor angiogenic vessels are either derived from endothelial progenitor cells or from existing vessels that propagate to feed growing tumors (15). The ECs present within the TME possess abnormal pericytes and pericyte coverage which enables leaks between tight junctions. This directly leads to the systemic circulation of tumor cells, i.e. presence of CTCs, thus increasing the tumor’s metastatic potential (16). Hypoxia triggers stromal release of VEGF. Subsequent activation of VEGF-2 receptors on adjacent ECs promotes their migration to the region of hypoxia and production of hypoxia-inducible factors 1 (HIF-1) and 2 (HIF-2) (17) ultimately leading to EC proliferation, migration and maturation (18, 19). The result is tumor endothelial anergy, the cellular non-response to pro-inflammatory stimulation (i.e. IFN-γ, TNF-α, and IL-1). As vital gatekeepers of the TME, tumor endothelial cells (TECs) are the primary barrier to immune-stimulatory cells which promote the loss of anti-cancer immunity (20–23), TME-derived cytokines such as VEGF, ET1, FGF-2, and EGFL7 function to inhibit tumor endothelial ability to upregulate the expression of chemoattractants (i.e. CXCL7, CXCL10, IL-6, and CCL2) and adhesion molecules (ICAM1 and VCAM1), consequently promoting immunosuppression and tumor progression (20, 23–26). Additionally, TECs were shown to promote regulatory T cell (Treg) accumulation *via* up-regulation of the lymphatic and vasculature endothelial receptor 1 (CLEVER-1); an abundance of CLEVER-1-positive macrophages support immunosuppression. It has been reported that tumor-induced CLEVER-1 expression in both macrophages and endothelial cell populations was required to support the growth of melanoma, and that the chief driver was the diminished expression of vascular E- and P-selectin, and accumulation of Tregs and M2 macrophages in the tumors induced by CLEVER-1 (27, 28).

ECs can selectively upregulate T cell inhibitory receptors including: IDO1, TIM3, B7-H3, B7-H4, PD-L1 and PD-L2 (29–32) along with other soluble inhibitory molecules such as: TGF-β, IL-6, IL-10, and PGE2 (33–35), thus maintaining a constant inflammatory state within the TME. ECs may also express apoptosis-inducing molecules such as TNF-related apoptosis-inducing ligand (TRAIL) and FasL which were shown to selectively extinguish effector T cells, sparing Tregs (36–40). Thus the tumor vasculature inhibits immune cell extravasation in the tumor bed and promotes the immunosuppressive state, and is one of the main modulators in immune resistance (41–43).

##### Mesenchymal Stem Cells

As important contributors to the TME, mesenchymal stem cells (MSCs) harvested from different tissues have demonstrated varying expression levels of factors that contribute to embryonic stem cell pluripotency, such as SOX-2, NANOG and OCT-4 (44, 45). MSCs are dispatched by a series of paracrine signaling pathways in response to injury, and either differentiate on-site to replenish damaged tissue with their cell multipotency (10, 46) or activate various trophic factors necessary to activate local SCs specific to the tissue (47), for the purposes of wound healing. The TME continuously recruits MSCs by generating constant inflammation, similar to that seen in wound healing, and is thus about to remodel itself perpetually (48, 49). Thus MSCs are able to populate the TME with other crucial cells such as pericytes and fibroblasts with their multipotency (50). In addition to the aforementioned, MSCs are involved in other cancer-promoting mechanisms. MSCs release specific molecules such as epidermal growth factors (EGFs) (51), IL-8/IL-6 cytokines (52) and CXCL1/2/12 chemokines (53) which directly act on cancer cells in a paracrine fashion and increase cellular proliferation by induction of phenotypic modification. In another immune suppressor mechanism, MSCs were shown to suppress both adaptive and innate immunity by directly inhibiting CD4 and CD8 T cell proliferation (54). A third mechanism includes stimulation of TLRs3/4 present on MSCs, inducing production of CXCL10, IL-8 and IL-6 which are crucial for T cell suppression (55). Furthermore, *via* adhesion to Th17 *via* CCL20, MSCs are capable of inducing T cell differentiation to Tregs thus suppressing both innate and adaptive immunity (56). MSCs also promote tumor revascularization by a) secreting various angiogenic factors such as EGF and VEGF, which are responsible for recruitment of ECs for vasculature maturation (57) or b) by converting into endothelial-like cells to modulate neo-angiogenesis (58). MSCs have also been shown to possess the ability to differentiate into tumor stromal progenitor cells, such as cancer-associated fibroblasts (CAFs), which further enhance the development and sustenance of the TME (59). MSCs have been demonstrated to be involved in the production of inflammatory chemokine CCL5, which is responsible for metastatic potential in breast cancer (60). MSCs are capable of impeding all immune responses through interactions with every cell in the immune system, directly or *via* soluble immune secretomes (40) such as:

prostaglandin E2 (PGE2) - PGE2 suppresses IL-2 formation and T cell function. The literature also suggests that PGE2 regulates the balance between different helper T cell (Th) configurations and responses, solely inhibiting Th1 IFN-γ production (61). PGE2-suppression of Th1 results from its ability to repress IL-12 production in dendritic cells (DCs), and monocytes (62, 63). Additionally, PGE2 is required for the development of myeloid-derived suppressor cells (MDSCs) (64) and tumor-associated macrophages (TAMs) (65). MDSCs express high levels of COX2, a major source of PGE2. The positive feedback between COX2 and PGE2 promotes MDSC stability, and leads to the production of additional MDSC-associated suppressive mediators (64). HIF-1-α also mediates and likely initiates a signaling cascade in PGE2-mediated MDSC development (66).

Indoleamine 2,3-dioxegynase (IDO) - Cells expressing IDO can suppress immunity by catabolizing tryptophan (Trp) and other indole compounds (67). Potent IDO inducers IFN-I and IFN-II are produced at sites of inflammation. IDO is also expressed by DCs, resulting in DC conversion to tolerogenic antigen-presenting cells (APCs) that suppress effector T cells (Teff) whilst promoting Tregs. Non-catalytic signaling induces TGF-β release by a subset of DCs, leading to tolerance. Tolerogenic IDO promotes tumorigenesis by allowing cancer cells to evade immune surveillance. Some cells express IDO1 genes which deplete Trp and generate bioactive catabolites such as kynurenines (Kyn). This is sensed by a population of immune cells, leading to suppression of innate and adaptive immunity (68).

Nitric oxide (NO) - a pleiotropic and short-lived radical which has pathophysiological functions. Produced by MSCs, NO is responsible for mediating T cell-dependent immunosuppression (69). MSCs express compounds such as arginase, β2 integral, Gr-1 granulocyte marker, and inducible nitric oxide synthase (iNOS) which converts L-arginine into urea and L-ornithine (70). This hints at a potential synergy between arginase and iNOS which would result in superoxide (O^2-^). O^2-^ then may react with NO to produce peroxinitrite (ONOO^-^) as well as other reactive nitrogen intermediates which induce T cell apoptosis (71). Another immunosuppressive mechanism is the high NO-concentration impairment of IL-2-R-induced signaling. This leads to the activation of Janus kinases 1 (JAK1) and 2 (JAK2), with signal transducer and activator of transcription factor 5 (STAT5) (72).

#### Cancer-Associated Fibroblasts

Cancer-associated fibroblasts (CAFs) produce key proteins such as periostin and Tenascin-C which are necessary for tumor support and metastasis (73, 74). Their expression in the TME changes the predominant cell type in the stromal tissue as well as the modulator of the ECM. It has been shown that CAFs placed with normal prostate cells *in vitro* induces rapid cell growth and alters prostate cell histology (75). The histological changes may be the result of CAFs’ ability to induce epithelial-to-mesenchymal transition (EMT) *via* upregulation of TGF-β, which modifies cellular cytoskeleton architecture, cyclin-dependent kinases, and decreases the potency of immune surveillance (76). This subsequently enables cellular migration and invasion, and induces the development of pluripotent tumor cells (77, 78). This is evident with the demonstration that growth factors such as CCL2 and hepatocyte growth factor (HGF) induced CSC renewal and stemness of cancer cells in both breast (79) and hepatocellular carcinoma (80). Another mechanism of stemness is the upregulation of the NF-κB signaling pathway. This prompts continuous secretion of pro-inflammatory cytokines such as IL-6 and IL-8; this constant inflammatory milieu induces EMT (81). The importance of IL-6 has been previously elucidated. Increased expression of IL-6 in myeloma cells induces activation of the JAK2-STAT3 pathway (82) and increases expression of Bcl-Xc which correlates with resistance to therapy (83). In early TME development, the ECM is reconstructed in a stiffened manner (84); the elastin component of the ECM is cross-linked with collagen in the presence of lysyl oxidase (LOX) and these are both produced by CAFs (85). CAFs are also responsible for the secretion of fibroblast growth factor-2 (FGF-2), an essential signaling molecule responsible for angiogenesis (86), and expresses stromal-derived factor-1 (SDF-1) which induces metastasis in breast cancer by acting as a chemotactic factor for circulating ECs (87). The role of Chi3L1, a non-enzymatic chitinase-3 like-protein 1, has also been studied. Regulated by the ECM, it binds to heparin, hyaluronic acid, and chitin, and is synthesized by a variety of cells including tumor cells, fibroblast-like cells, smooth muscle cells, chondrocytes, macrophages, neutrophils, and synoviocytes (88). Genetic targeting of CAF-derived Chi3L1 in fibroblasts has attenuated recruitment and reprogrammed macrophages to an M2 phenotype, which promotes a Th1 phenotype in the TME (89). Additionally, the TAM polarization to the M2 phenotype was shown to be induced by high expression of TGF-β (90). In an *ex vivo* model of oral squamous cell carcinoma, CAFs promoted the development of an M2-like phenotype from CD14 myeloid cells after induction by IL-10, TGF-β, and ARG1. This ultimately suppresses T cell proliferation (91). With their reciprocal interactions, TAMs and CAFs are central immunosuppressive players in the TME. Notably, CAFs recruit macrophages through the expression of stromal cell-derived factor 1 (SDF-1/CXCL12). SDF-1 magnifies M2 polarization of macrophages mirroring high production of immunosuppressive cytokine IL-10 (92–94).

#### Pericytes

Arising from differentiated mesenchymal precursors, pericytes are recruited when cancer cells overexpress platelet-derived growth factor beta (PDGF-β) (95) in both healthy and neoplastic tissues alike. They exhibit many tumor-supporting mechanisms including the release of EC-attracting soluble factors, which rapidly induces revascularization of the TME (96). In addition to their angiogenic properties, pericytes express the cluster of differentiation (CD) markers of MSCs. Their potential for multipotency contributes to metastatic processes by generating other stromal cells for the TME (97). Furthermore, pericytes have been shown to induce immune suppression through secretion of various soluble factors including prostaglandin E2 (PG-E2), TGF-β and nitric oxide (98). Pericytes are capable of regulating T cell trafficking and modulation. Pericytes of the TME were shown to express PD-L1 and PD-L2, responsible for T cell exhaustion (99). Retinal pericytes, too, exhibit immunosuppressive properties. When pericytes were cultured with activated T cells, production of IFN-γ and TNF-α decreased. Pericytes coexpressing CD248, CD90, and PDGFR isolated from human gliomas were able to inhibit cytotoxic T cell (CTL) proliferation, and thus induce immunosuppression within the TME (98, 100). Additionally, pericytes from normal brain tissue and malignant gliomas secrete immunosuppressive factors such as: PGE2, TGF-β, and NO, previously shown to inhibit anti-tumor response and suppressed mitogen-activated T cell activity (98). Pericytes produce growth factors, chemokines, cytokines, and adhesion molecules which regulate the microenvironmental ability to evade immune surveillance.

#### Cancer Stem Cells

The majority of cancer cells arise from cancer stem cells (CSCs) that express surface markers similar to that of stem cells (SCs), such as CD44, CD90 and CD133. It is uncertain whether CSCs arise from non-SCs or from somatic SCs (101, 102). The tumorigenic potential of CSCs was shown when leukemia-initiating SCs from AML patients were transplanted into severe combined immunodeficiency (SCID) mice, which later developed AML (103). In another study, CSCs and a non-CSC counterpart were injected into immunodeficient mice; only the CSC-injected mice were able to repopulate parental tumor cells (104). The theory of CSC is further supported by their discovery in breast, brain, colon, hematopoietic and lung cancer (101, 105). As the architects of the TME, CSCs are able to self-renew and drive the pathophysiologic mechanisms of carcinogenesis aided by various non-cancerous cells (101). CSCs possess both plasticity and immunomodulatory features capable of evading immune surveillance, thus they are the most distinguished malignant cell unit implicated in primary cancer or in resistance to immunotherapy. Bidirectional release of cytokines, cell-to-cell communication *via* extracellular vesicles, and fusion of CSCs with fusogenic stromal cells are mechanistic immunomodulatory properties of CSCs. Recent studies suggest that CSCs are pivotal players in immune escape: due to their immunomodulating nature, they are capable of cellular dormancy whilst evading immunosurveillance (106, 107). The tumor niche consists of intratumor immune cell populations which interact with CSCs and affect their functional status (108, 109). Undergoing cell-to-cell fusion (a process which generates tumor cell hybrids under pathological conditions) with various sorts of microenvironmental fusogenic cells such as: fibroblasts, macrophages, MDSCs and MSCs, the tumor niche contributes to the formation of aberrant cells that possess SC-like properties and are correlated with tumor initiation, progression, and metastatic potential (110, 111). CSC-related immune escape mechanisms are further complicated by epigenetic perturbations (112). Epigenetic modifications of differentiated cancer cells and CSCs can lead to expression modifications in immune-related genes. This domino effect impacts antigen presentation, processing, and immune evasion. For example, re-expression may be possible through demyelinating agents, allowing for immunotherapeutic applications (113). CSCs contribute to metastasis and tumor heterogeneity, implying their capacity for resistance to chemo-, radio-, and immunotherapies, and more besides (114). The principal limitation of efficacious anti-CSC treatment is the challenge in recognizing CSC-characteristic biomarkers.

#### Immune Cells

With regards to carcinogenesis, immune cells possess dual action dependent upon various chemokine expressions within the TME. It has been previously shown that Tregs, M2 macrophages and T-helper 2 cells (Th2) support tumorigenesis while NK cells, antigen-presenting cells (APCs), cytotoxic T cells (Tcs, CTLs) and M1 macrophages are protective against tumor development. High expression of chemokines such as CXC (1-16), with their respective CXC receptors (CXC-R), attracts various cancer-supporting immune cells that have been shown to be responsible for poorer prognosis in colorectal cancer (115).

Macrophages possess critical phagocytic properties in the adaptive and innate systems. Tumor-associated macrophages (TAMs) are derived from CCR2 inflammatory monocytes, and are classified as either pro-inflammatory (M1) anti-cancer cells through the production of IL-1 and tumor-necrosis factor alpha (TNF-α) (116) or anti-inflammatory (M2) cancer-supporting cells through the production of immunosuppressive cytokines such as IL-10 (117). M2 macrophages have been linked to progression in colon, renal cell and breast carcinomas (118–120) *via* multiple mechanisms. Primarily, anti-inflammatory cytokines and chemokines cause immune suppression by inhibiting T cells and NK cells (121, 122); chemokines CCL5, CCL20 and CCL22 recruit Tregs and activate their inhibitory actions *via* production of IL-10 and TGF-β1 (123). Secondly, angiogenesis is induced by the release of signaling protein WNT7B, which targets ECs for stimulation of VEGF (124). This produces another major angiogenic factor called pro-matrix metalloproteinase 9 (proMMP9) (125). M2 macrophages also facilitate carcinogenesis and metastasis through the production of CCL18 (126) and the nuclear factor- κB/FAK pathway (127) leading to induction, migration, invasion, and the EMT. Lastly, TNF is a product of both activated macrophages and the cells of the TME; in addition to being an anti-cancer cytokine, it has been implicated in the inflammatory process necessary for tumor growth (128). The role of STAT3 as a mediator between TAMs and tumor cells has been elucidated, showing that STAT3 activation inhibited Th1 subtype differentiation by blocking the expression of immune-stimulatory mediators (129).

Like TAMs, tumor associated neutrophils (TANs) demonstrate two subtypes: the N1 TAN phenotype which possesses anti-tumor action, and the N2 TAN phenotype which has tumor-support activity (130). Sustained inflammation induces an IL-8-dependent neutrophil chemotaxis within the TME (131). As previously described, TGF-β was shown to be highly expressed within the TME, inducing a generalized immunosuppressive state; additionally it was shown to polarize TANs into the N2 phenotype (130). N2 TANs sustain inflammation within the TME by releasing genotoxic elements such as NO and ROS (131). Tumor models have shown that N2-TAN-mediated immune suppression was achieved through various mechanisms: 1) production of TNF-α and NO to induce T cell apoptosis (132); 2) inhibition of T cell proliferation through modulation of PD-1/PD-L1 signaling and release of arginase (133); 3) N2-TAN expression of TGF-β, and 4) production of CCL17, shown to recruit Tregs to further induce an immunosuppressive state (134).

T cells, part of the adaptive immune system, prevent tumor growth through lytic action and the production of IFN-γ-dependent cell-cycle arrest (135). After lysis, the cell component is phagocytosed and expressed on APCs, exposing them to maturing lymphocytes and resulting in tumor suppression (136). Tregs impede the immune response by expressing various cytokines against anti-tumor cells. It has been shown that when the Treg-to-CD8 ratio is high in hepatic (137) and breast carcinoma (138), this results in uncontrolled progression and worse prognosis. Th2 is yet another cell responsible for promoting the necessary inflammatory state within the TME, and it has since been proposed as an agent in tumor progression. Countering the anti-tumor activity of Th1, Th2 has been associated with poorer prognosis when detected (128). Its differentiation is driven by thymic stromal lymphopoietin (TSLP), an IL-17-like cytokine produced in response to TNF-α and IL-1-β from TAMs and TME stromal cells (139).

APCs are innate cells which process and display antigens bound to major histocompatibility complexes (MHCs) to naïve T cells to induce cytotoxicity. They are categorized into professional (dendritic cells; DCs) and non-professional (fibroblast) APCs. It has been previously shown that because of the presence of IL-6 and granulocyte-colony stimulating factors (G-CSF), APCs of the TME lack the co-stimulatory receptor B7 and cannot stimulate T cell cytotoxicity. This alters the differentiation of APC to mature cells (140). Additionally, various signals within the TME induces differentiation of granulocytes to immunosuppressive cells such as TAMs and tumor-associated neutrophils (TANs) (141).

NK cells are an important innate component responsible for destroying tumor cells and preventing the progression of tumorigenesis. In the immunocompetent, NK cells select out the APCs with improper expression of MHC-I and retain a pool of competent APCs (142). However NK activation is greatly inhibited within the TME due to excess production of TGF-β and other anti-inflammatory cytokines and chemokines (143). Microarray analysis of extra-tumoral and intra-tumoral NK cells in the lung tumor microenvironment demonstrated upregulation of cytotoxic gene expression, and intra-tumoral NK cells were associated with better prognosis (144).

B cells are most common in draining lymph nodes. They have been shown to infiltrate tumor margins and have been associated with proper antibody response in ovarian and breast carcinomas (145, 146). On the other hand, B cells have been shown to differentiate into another tumor-associated cell. An IL-10-secreting B cell named Breg (147) promoted metastasis of breast cancer (148) and it has been shown to be implicated in inflammation-induced squamous cell carcinoma through the secretion of TNF-α in animal models (149). It should be noted that this B cell was non-infiltrating – that is, present only in the surrounding tissue – thus further studies are warranted to determine if they behave the same way in human cancers.

Little is known of myeloid-derived suppressor cells (MDSCs). They are identified as immature myeloid cells that are upregulated in cancer and other inflammatory processes (150). Their phenotype is variable and their characterization is difficult. MDSCs can also differentiate into TAMs, as they both possess immunosuppressive markers such as CD115 and F4/80 (151). It has been shown that MDSCs are able to directly suppress CD8 cells by producing nitric oxide synthase-2 (NOS-2) and arginase (ARG-1) (71). Another immunosuppressive mechanism exhibited by MDSCs is their positive effect on T cell differentiation into cancer-supporting Tregs (152).

Dendritic cells (DCs), the so-called professional APCs, are among the first cells to appear during inflammatory states. Varying subsets of DC maturation have been observed in the TME (153); this typically comprises of only a few mature DCs and is associated with better prognosis (154). Generally, the previously described immunosuppressive states impair DC maturation and activation (155). As stated, the DC maturational stage is crucial for normal function. Multiple subsets have been identified including anti-tumor classical DCs with high CD8 and NK cell-activation activity (156); while plasmacytoid DCs (157, 158) and monocyte-differentiating DCs have either immune-supportive or immune-suppressive actions (153, 159). The known immune suppressor PD-L1 is highly expressed within the TME. Tumor derived factors directly increase the expression of PD-L1 in DCs and MDSCs, further inhibiting anti-tumor immunity (160).

#### Cancer-Associated Adipocytes

Adipocytes are known, key contributors to the TME and are proposed to be involved in the metastatic process, angiogenesis, and resistance to apoptosis (161). Cancer-associated adipocytes (CAAs) are a broad grouping of the following: intratumoral adipocytes, peritumoral adipocytes, recruited adipocytes, and *de novo* differentiation of MSCs into adipocytes or adipocyte-like cells that store large amounts of energy-rich lipids (162). It has been shown that mature adipocytes incubated with breast cancer cells induced phenotypic change of adipocytes into fibroblast-like cells that contributed to the expansion of CAFs, well-known immune suppressors (163). CAAs can influence the TME through direct contact with adjacent cells or in a paracrine manner through the production of adipokines, hormones and proinflammatory cytokines (i.e. CCL6, CCL2, CCL5, MMP, VEGF, TNF-α, insulin and leptin, to name a few) to facilitate cancer invasion and immune resistance (164). CAAs were shown to possess dysfunctional proinflammatory features that support the TME (165). CCL2 and CCL5 released from CAAs were shown to recruit and promote M2 polarization of macrophages (166). Furthermore, the high concentration of TNF-α and IL-6 mediated JAK2/STAT3 pathway activation to induce phenotypic change into breast cancer cells with SC properties (164). The important adipokine leptin was also shown to make use of the JAK-STAT3 pathway to induce cancer stemness and evade immune surveillance (167). In cachectic mice, phenotypic change in white adipocytes with overexpression of uncoupling protein 1 (UP-1) induced their differentiation into brown adipocytes with fibroblastic characteristics (168). Furthermore, signaling proteins within the TME (i.e. IL-6, exosomal contents, and parathyroid hormone related peptide PTHrP) were shown to promote phenotypic variations into brown adipocytes (169). Because PD-L1 is strongly expressed on brown adipocytes, PTHrP has been linked to tumor invasion and metastasis. Phenotypic variations leading to the differentiation from white to brown adipose tissue appears to be another immunosuppressive mechanism (170).

### Extracellular Matrix

The extracellular matrix (ECM) contributes the largest component of the TME and is composed of proteins such as collagen, proteoglycans, hyaluronic acid and laminins (171). The ECM is crucial for the maintenance of the TME and the induction of metastasis. Aside from acting as a physical cellular scaffold, it is responsible for cellular adhesion and migration out of the TME. It stores various soluble factors such as angiogenic factors and chemokines that induce a continuous inflammatory state, resulting in expansion of the cellular repertoire (172). The continuous inflammatory state exacerbates the conversion of stromal fibroblasts into myofibroblasts (173) which in turn deposit large amounts of growth factors and ECM proteins, inducing contraction and increasing stiffness (174). Newly-deposited ECM proteins are acted upon by CAF enzymes such as LOX to further stiffen the ECM; stored growth factors are subsequently released to amplify the circuitry between the tumor cells and its ECM. This eventually contributes to metastasis and ensures ECM resistance to treatment (175). The ECM can influence the recruitment of immune cells into the TME. For instance, the ECM can drive PI3K/AKT (pro-survival pathway) activation, which facilitates CSC immune evasion (176). ECM proteins can also recruit immunosuppressive cells such as Tregs and TAMs which were shown to promote CSC survival while blocking anti-tumorigenic immune cell (i.e. CTL) recruitment (177–179). The ECM is capable of impairing the proliferation and activation of T cells, which are responsible for eliminating CSCs (180). The composition of the ECM also plays a crucial role in modulating the state of tumor infiltrating immune cells. For example, M2 polarization of macrophages is achieved in a periostin-rich or stiff collagen-rich ECM (181). After recruitment, CSC survival signaling pathways such as Src, STAT3/SOX2, Hedgehog, and NF-κB are activated by the M2 macrophages, leading to inhibition of T cell proliferation and activation through type I collagen-dependent fusion of LAIR receptors while sequestering T cell proliferation growth factors (177). In addition to the aforementioned, neutrophils and TAMs are capable of selectively recognizing the EMC in order to promote cancer growth as they are recruited to the microenvironment (182, 183). This implies the ability of the ECM to modulate immune surveillance in the CSC microenvironment.

An increase in metabolic stress and hypoxia leads to poor diffusion in ECM-rich tumors, ultimately up-regulating immunosuppressive factors such as: CCL22, CCL18, TGF-β, IL-10, VEGF-B, and PGE2 (184–186). TGF-β in particular acts as a suppressor of CD8 CTLs and NK cells in the TME by attracting Tregs and functioning as an M2-polarizing agent for macrophages (186–188). Both of these phenomena negatively regulate infiltration and activity of CTLs (189). In addition, T cells are suppressed by VEGF-A which recruits Tregs that express NRP1 (a coreceptor of VEGF) (190, 191).

### Micro-RNA Deregulation

Micro-RNAs (miRNAs, miRs) are an endogenously-expressed class of non-coding single-stranded RNA fragments that are involved in gene expression modulation. By targeting mRNA at the post-transcriptional level, they may act as tumor suppressors or oncogenes (192). When deregulated they are associated with tumorigenesis and metastatic development (193). Oncogenic miR-21 overexpression in CAFs has been associated with tumor aggressiveness *via* induction of angiogenesis and treatment resistance (194, 195). MiR-155 and miR-210 over-secretion in cancer cells has been shown to induce the transition of MSCs and normal fibroblasts to CAFs, thus reinforcing the TME (196, 197). Overexpression of miR-17-to-92 may lead to downregulation of tumor suppressor genes, much like with oncogenes, inhibiting apoptosis *via* various pathways (198). Other types of miRNAs include the tumor suppressors comprising of miR-126, whose main function is to suppress MSC recruitment in the TME by inhibiting SDF-1 and CCL2. When downregulated, miR-126 has been shown to promote breast cancer metastasis by inducing fibroblast recruitment and EMT (199). MiRNAs represents another hurdle to consider when evaluating TME defenses. Cancer cell-derived immune modulatory miRNAs regulate a multitude of immune components such as CTLs, Tregs, NK cells, DCs, and MDSCs *via* intracellular communication (i.e. micro vesicles and exosomes). Cancer-derived miRNAs have been implicated in various mechanisms to induce immune evasion. This is achieved through the modulation of expression profiles using histone modification and DNA methylation (200). It has been shown that these epigenetic pathways occur simultaneously and act on each other, i.e. DNA methylation- or histone acetylation-induced deregulation of miRNAs, and vice versa (201, 202).

#### miRNAs: Modulating Antigen Processing and Presentation in Cancer

A number of miRNAs interrupt MHC-I and antigen-processing machinery (APM) components in cancer cells (**Table 1**). A study of nasopharyngeal cancer cells indicated that miR-9 targeted a multitude of APM constituents such as β2-microglobulin, low molecular weight polypeptide subunits LMP10, LMP9, LMP8, and transporter associated with antigen processing 1 (TAP1). MiR-9 has the potential to downregulate MHC-I molecules (i.e. HLA-H, HLA-B, HLA-C, and HLA-F) and its overexpression in cancer cells enhances immune tolerance in the TME (203). MiR-346 is an endoplasmic reticulum (ER) stress-associated miRNA which regulates the immune response by indirectly suppressing the IFN by targeting adenylate uridylate-rich elements (AREs) on the 3’-UTR region of mRNA transcripts resulting in termination; or by directly phosphorylating TAP1, resulting in interference of MHC-I signaling pathways (206, 207). Likewise, miR-125a-5p in esophageal adenocarcinoma cells bind to the 3’-UTR of TAP2 mRNA resulting in interference with antigen presentation (204). Proteomic screening of miR-27 decreased cell surface expression of MHC-I expression, promoting cancer progression; miR-27-a-induced MHC-I downregulation depended on calreticulin suppression (an essential calcium-binding protein which regulates gene transcription) (208).

**Table 1:** Cancer antigen processing and presentation, regulated by miRNAs.

Cancer cell type	miRNA	miRNA target	Reference
Nasopharyngeal cancer	miR-9	β2-microglobulin	(203)
Nasopharyngeal cancer	miR-9	LMP9/10	(203)
Nasopharyngeal cancer	miR-9	LMP8	(203)
Lung cancer	miR-451		
Nasopharyngeal cancer	miR-9	TAP1	(203)
Esophageal adenocarcinoma	miR-125a-5p	TAP2	(204)
- Nasopharyngeal cancer	miR-9	MHC-I	(203–205)
- Esophageal adenocarcinoma	miR-148a-3p		
- Colorectal cancer	miR-27a		

LMP, low molecular weight polypeptide subunit; TAP, transporter associated with antigen processing; MHC-I, major histocompatibility complex class I.

#### miRNAs Targeting HLA-G

This non-classical MHC-I molecule has immune inhibitory function, and it can be hijacked by cancer cells to escape immune attack. When HLA-G binds to NK cells and CTLs, the effector cell cytotoxicity is suppressed (209). HLA-G expression is elevated in cancers such as endometrial, breast, melanoma, gastric, hepatocellular, lung, and colorectal carcinoma (210, 211). The increase in HLA-G expression correlates to the loss of regulatory HLA-G-targeting miRNAs such as miR-152, miR-148a, and miR-148b (212, 213). For instance, the oncogenic estrogenic G-protein-coupled estrogen receptor 1 (GPER) signaling pathway is known to decrease miR-148 levels in breast cancer cells, contributing to cancer immune evasion (214).

#### miRNAs Associated With Immune Checkpoint Ligands

Immune checkpoint signaling is determined by factors including pre-existing inflammation of the oncogenic signaling pathway. Studies indicate that an increase in PD-L1 expression on numerous cancer cells was achieved by a loss of miR-138, miR-34a, miR-191-5p, miR-148-3p, miR-873, miR-479-5p, miR-195-5p, and miR-3609. A decrease in miR-383 was shown to profoundly elevate PD-L1 expression on cervical cancer cells (215–220). In contrast, PD-L1 expression is promoted by miR-18a *via* SOX6, WNK2, and PTEN signaling pathways. After induction of PD-L1 expression, various pathways (Wnt/beta-catenin, ERK, and PI3K-AKT) were activated, ultimately leading to PD-L1 transcription (221).

#### Phenotypic Variations Induced by miRNAs

MHC-I chain-related molecule A and B (MICA, MICB) (222), and UL16-binding protein (ULBP) are stress-induced ligands which are recognized by the presence of NKG2D present on CTLs and NK cells (223). NKG2D is responsible for maintaining cancer immune surveillance, and is downregulated in cancer cells, resulting in cancer cell immune escape at the post-transcriptional level (224). Alternatively, it has been shown that various miRNAs directly target the ULBP2 3’-UTR, and overexpression of these miRNAs lead to downregulation of ULBP expression. Such miRNAs include miR-34a, miR-34c in malignant melanoma and miR-519a-3p in breast cancer (202, 225).

MiRNA mimics can inhibit receptor expression hereby diminishing tumor cell recognition by NK cells. MiRNAs function at the post-transcriptional level of gene expression, and both miRNAs and IFN-γ downregulate expression of the MICA ligand. MiRNA targets MICA/B mRNA by directly binding to the 3’UTR of the target gene, causing mRNA degradation or translation repression. MiRNAs that target MICA include miR-93, miR-106b, miR-106a, miR-373, miR-20a in hepatocellular carcinoma (HCC), miR-519a-3p, miR-20a in breast cancer, and miR-125b in multiple myeloma (202, 226, 227). MiRNAs that target MICB include miR-20a in breast cancer (228), and miR-302c and miR-520c in multiple cancers (228, 229). This leads to immune escape of malignant cells.

#### miRNAs Relative to Cancer Cell Metabolites

Tryptophan (Trp) is an example of a metabolite responsible for maintaining the function of tumor infiltrating lymphocytes (TILs). The rate limiting enzyme of Trp metabolism, converting Trp to 3-hydroxyanthranilic acid and kynurenine, is IDO1 (230). Increase in IDO1 expression with concurrent decrease in Trp leads to dysfunctional Teffs, permitting cancer immune evasion (231). The downregulation of miR-218 and subsequent elevation of IDO1 has been shown to safeguard cervical cancer cells from immune attack (232).

#### Cancer Cell-Derived miRNAs Which Regulate Immune Evasion Through Vehicles or Exosomes

Cancer-derived miRNAs are capable of exhibiting extracellular bio-activities through microvesicles or exosomes as well as modulate the expression profile within cancer cells (233). Cancer-derived miRNAs can be transferred *via* exosome to several TILs in order to mold an immunosuppressive microenvironment. CAFs are immune cells regulated by cancer cell-derived exosomal miRNAs. They can be reprogrammed by various miRNAs to induce tumor progression (234). MiRNAs released into the TME by CAFs function as paracrine stimuli for the activation of adjacent fibroblasts and cancer cells. Exogenous overexpression of miRNAs leads to fibroblast-to-CAF-like cell conversion, resulting in immune suppression (235). Some examples of CAF-derived miRNAs include miR-21 and miR-1247-3p in HCC, and miR-27a in gastric cancer (236–238).

Notably, MDSCs are another class of immune cells which are regulated by cancer cell-derived exosomal miRNAs; miR-17-5p (breast cancer) and miR-20a (in several cancers) (239, 240) promote the STAT3-mediated suppressive function of MDSCs. Additionally, miR-21 and miR-155 show associations with STAT3 activation through the phosphatase and tensin homolog (PTEN) target, along with SHIP1, leading to MDSC expansion in both granulocytic and monocytic subpopulations (241).

TAMs are also immune cells derived from exosomal miRNAs, and can be activated through two pathways: M1 (classical pathway), and M2 (alternative pathway); two perform different regulatory functions in the TME (242). Several miRNAs engage in the polarization into M2 macrophages, which inhibits immune surveillance. For instance, miR-21 regulates TAM through IFN-γ/STAT1 and PTEN to promote M2 polarization, increasing tumor cell migration while decreasing PD-L1 expression and M1 polarization (243, 244). Additionally, miR-324 in colon cancer targets CUEDC2, which regulates TAM to increase pro-inflammatory cytokine production as well as further increase tumorigenesis (245).

### Exosomes

These extracellular micro-vesicles contain components of proteins, lipids and genetic materials of the parent cell (246), and are potent signaling molecules within the TME. Exosomes arising from both malignant and non-malignant cells have been shown to be involved in tumorigenesis, therapy resistance, and immune resistance (247).

Homotypic transfer of exosome refers to signal transfer between cancer cells. Glioblastoma cell exosomes were shown to induce change in wild-type cells *via* transfer of the oncogenic protein epidermal growth factor receptor 3 protein (EGFR-v-III) (248). Similarly, it has been shown that exosomes from breast cancer cell lines and breast cancer patients, which contain miRNA machinery, were able to induce malignant transformation in normal cells (249). Another study showed that exosomes arising from pancreatic adenocarcinoma were able to modify the NOTCH-1 pathway and inhibit cellular death (250). Homotypic exosome transfer promotes cancer progression *via* the oncogenic pathway.

Heterotypic transfer of exosome, as previously described with regards to tumor growth and dissemination, is widely dependent upon its TME. Cellular crosstalk between the TME and either internal or external components is crucial for TME survival; this is achieved through multiple signaling networks such as paracrine and juxtacrine pathways (251). A study was conducted to spatially separate the TME. The complexity of the system was observed, with the authors demonstrating a vast cellular heterogeneity that consisted of six interacting layers of cells (252). Heterotypic transfer of exosome not only supports tumor growth but also elicits cellular resistance to various therapies as well as the harsh conditions within the TME (247).

### Cancer Cells

Aside from the immunosuppressive TME, cancer cells themselves when exposed to CTLs were shown to evade immune surveillance by modifying intrinsic mechanisms. These include expression downregulation of tumor associated antigens (TAAs), expression upregulation of PD-L1/2, and mutation induction within the antigen-binding machinery (β2-macroglobulin and HLA) and extrinsic pro-apoptotic genes such as CASP8 (253, 254). In addition, it was recently shown that clonal expansion of TAAs strongly correlated with the intensity of the immunogenic response (255). In analyzing the tumor genomic landscape, two mechanisms for TAA loss were observed:

1-immune-mediated elimination of TAAs presented by immune cells, followed by outgrowth of the remainder following “Darwinian evolutionary theory”;

2- acquisition of one or more genetic alterations, resulting in TAA loss and subsequent expansion of resistant clones (256). In determining how the EMT may contribute to immune escape, a study demonstrated that after prolonged exposure of breast carcinoma cells to CTLs, expression to TNF-α or *via* stable expression of SNAIL was increased. Protection from CTL-mediated lysis was linked to the activation of the autophagy pathway, which led to the survival of cells through dormancy (257). Impairment of CTL-mediated lysis was evident in another study in which breast carcinoma cells elicited increased TGF-β expression by silencing the Wnt1-inducible signaling pathway protein 2 (WISP2), which resulted in stemness (258, 259). Autophagy was not evident in the resistant phenotype; however inhibition of TGF-βwas able to induce EMT reversal thus rendering cancer cells more sensitive to CTLs (260). This suggests that chief developmental pathways utilizing TGF-β are fundamental in mediating immune resistance to CTLs. It is evident that along the EMT spectrum, several mesenchymal cancer cell variants have the potential to engage further mechanisms of resistance.

Tumor hypoxia is a significant parameter, as a driver of the EMT, tumor immune escape, and heterogeneity (261). Cells derived from a lung adenocarcinoma model were induced by hypoxia, and demonstrated a shift towards mesenchymal phenotypes. Only some cells underwent the EMT thus promoting cancer cell heterogeneity (262). Hypoxic stress leads to the emergence of cancer subclones, and analysis of these cells showed an increased tendency to resist CTL-mediated lysis. Of note, the resistance mechanism is suspected to be independent of E-cadherin-CD103 interaction. This is because TGF-β inhibition minimized cellular resistance to CTL-mediated killing without causing any changes to the E-cadherin expression in mesenchymal cancer cells (262).

CTLs primarily utilize the perforin/granzyme pathway to demolish target cells. When the perforin pathway is activated, further counter-mechanisms such as Fas or TRAIL are engaged at the cancer cell surface to induce T cell apoptosis (263). The pancreatic carcinoma model was used and given an EMT inducer in the form of the novel tumor antigen Brachyury. The cancer cells showed decreased susceptibility to CTL-mediated killing compared with control. Target cancer cells were co-cultured with CTLs, and poor killing was observed under experimental conditions. This was due to defective caspase-dependent apoptotic cell death despite immune antigenicity (264, 265).

Additionally, defects in the APM – correlated to immune-proteasome deficiency – was found to be common among cancers with a greater mesenchymal profile, and ultimately affected T cell-mediated cytotoxicity (266). Manipulation of cell-to-cell interactions and immunological synapses (IS) has been linked to immune resistance.

The IS involves interactions between immune killer cells (NK cells and CTLs) with their APCs or targeted cancer cells necessary to achieve maturation and production of TNF-α and IFN-γ, and their lytic functions (267–269) IS formation in T cells is regulated by cytoskeletal elements (i.e. actin), interaction of MHC-TCR, and the integration of integrin-based signals, generated when integral molecules (lymphocyte function-associated antigen 1, LFA-1) on the T cell interact with ICAM-1. Integrins undergo conformational changes through phosphorylation cascades (i.e. phosphotyrosine kinase activation linking integrins to the actin cytoskeleton) during peptide-MHC/TCR ligation. The actin cytoskeleton polymerizes at the edges of the active synapses, causing an increase in synaptic diameter size and immune cell flattening (270). This phenomenon leads to the emergence of T cell receptor (TCR) microclusters. These clusters merge at the center of the IS zone and are referred to as the central supramolecular cluster. In contrast, microclusters found at the periphery of the synapse join to form a highly contractile zone called the peripheral supramolecular activation cluster (271).

Mechanical forces brought about at the synapse *via* intercellular adhesion also play a role in rearranging the actin cytoskeleton and regulating adhesion-based signals. IS and its relationship with NK cells abide by similar rules, differing only in that NK cells express 2B4, DNAM1 and NKG2D receptors, rather than TCR. These receptors also regulate signaling activity and the changes in the integrin-actin network at different points of NK cell cytotoxicity. Numerous genetic aberrations have been shown to alter various stages in CTL and NK cell cytotoxicity, F-actin/microtubule networking, and cellular recognition which ultimately leads to NK or T cell disorders, resulting immunodeficiency (272, 273). These examples highlight the role of the operational IS in an appropriate and effective immune response.

The establishment of the IS and activation cascades relies on heterophilic interactions between ICAM-1 and integrins on target cells; the loss of ICAM-1 can be expected to impede IS formation. Moreover, manipulation of the actin network through changes in mechanical forces renders a significant effect on the IS and the lytic commitment (274, 275). The discomposure of the actin network in certain cells will either render them more resistant or more susceptible to CTL-mediated lysis (276).

**Figure 1:**
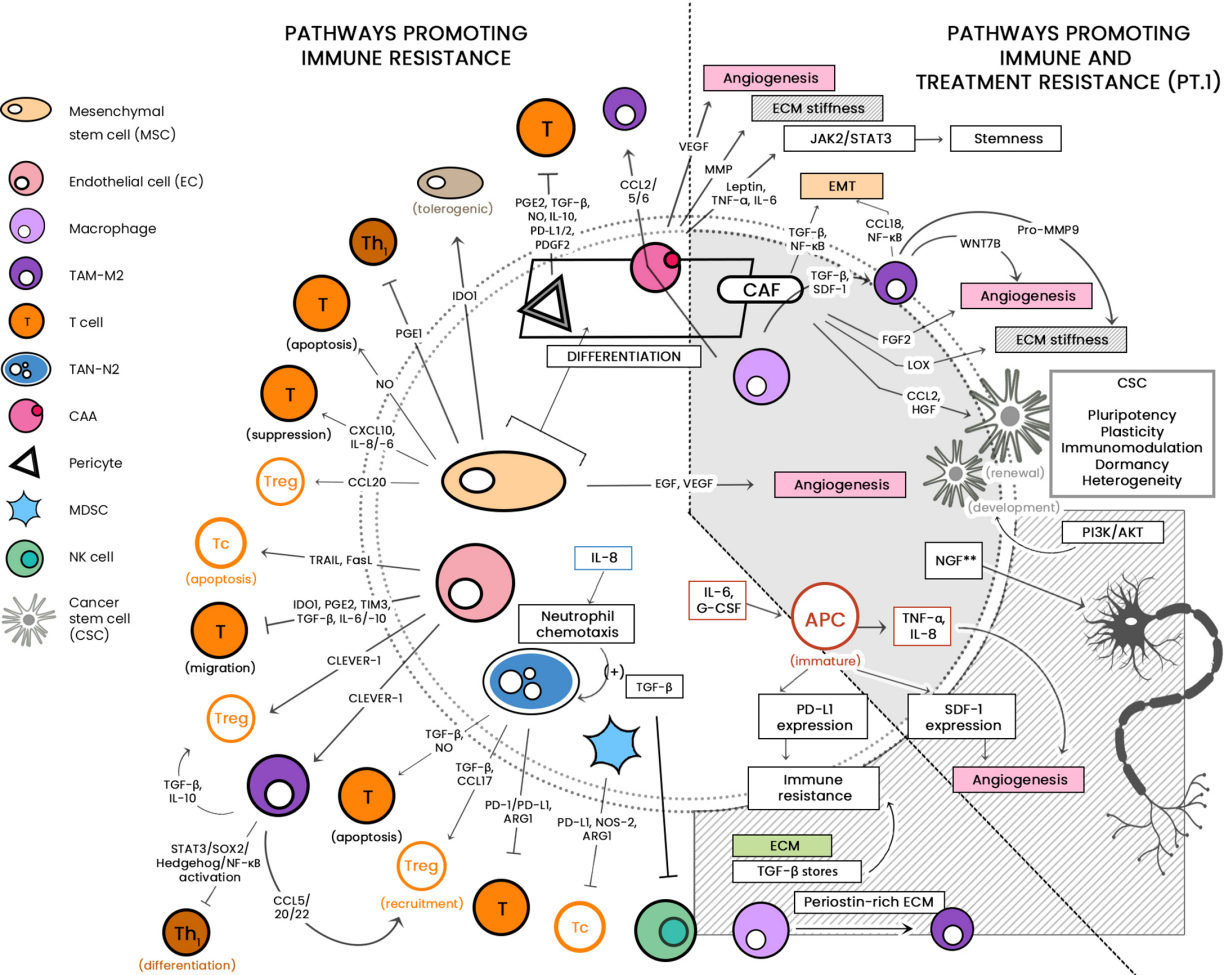
The large cellular repertoire of the tumor microenvironment (TME) is depicted in this diagram. Through the release of soluble factors, the presented cellular entities are seen to be involved in: 1) immune suppression, by either inducing apoptosis or inhibiting anti-tumor activity; and 2) both immune/drug resistance by stiffening the extracellular matrix, inducing epithelial-to-mesenchymal transition (EMT) and induction of stemness. CCL, C-C motif chemokine ligand; PG-E2, Prostaglandin E2; TGF-ß, Transforming growth factor beta; NO, nitric oxide; IL, interleukin; IDO-1, indoleamine 2,3-dioxygenase 1; TRAIL, TNF-related apoptosis-inducing ligand; Fas-L, Fas-ligand; TIM-3, T-cell immunoglobulin and mucin-domain containing-3; CLEVER-1, lymphatic endothelial and vascular endothelial receptor-1; PD-1/-L1, programmed cell death protein 1/ligand 1; Arg-1, arginase; NOS, nitric oxide synthase; JAK/STAT, Janus kinase/signal transducer and activator of transcription; ECM, extracellular matrix; CSC, cancer stem cell; PI3K/AKT, phosphatidylinositol 3 kinase/protein kinase B; NGF, neurotrophic growth factor; TNF-α, tumor necrosis factor alpha; SDF-1, stromal-derived factor-1; EGF, epidermal growth factor; VEGF, vascular endothelial growth factor. **associated with treatment resistance; mechanism as-yet-unknown.

See **Figure 1** for a diagrammatic summary of the major pathways that promote immune resistance, and immune and treatment resistance.

## Characteristics of the TME and Its Effects on Treatment Resistance

### The Tachyphylactic TME

#### Epigenetics: The Link to Treatment Resistance

To unleash, hijack, and restrict cellular plasticity, CSCs play a chief and fundamental role in epigenetics. In cancers, one of the most habitually mutated gene classes are epigenetic regulators, resulting in this characteristic uncontrolled cellular self-renewal. Epigenetic regulator mutations lead to oncogenic cellular reprogramming during cancer initiation. CSCs are either promoted or inhibited by the epigenetic mechanisms that integrate the cell-extrinsic (microenvironmental signaling), or cell-intrinsic (subclonal mutations) effects that establish intratumoral heterogeneity. Over time, CSCs generate self-renewing subclones with diverse fitness, whilst environmental changes are able to act on their genetic heterogeneity and modulate their phenotype. Further discussion on the CSC mechanistic roles and implications now focuses on how cellular plasticity can be affected by manipulation of DNA methylation and chromatin. In addition to the previously described role of miRNA, the following sections will shed light on epigenetic DNA methylation and histone modification leading to the development of CSCs, followed by the role of CSCs in drug resistance (277).

### Pathways Involved in CSC Development

#### Wnt/β-Catenin Signaling Pathway

β-catenin is transcription co-activator regulated by the WNT gene family, and is mainly involved in embryonic development, adult homeostasis, and, if highly expressed, various cancers (278, 279). Physiologically, the absence of WNT signaling keeps β-catenin at low levels through the ubiquitin-proteasome system (UPS). β-catenin is recruited into a destruction complex consisting of the adenomatous polyposis coli gene (APC gene) and Axin. This promotes the phosphorylation of β-catenin by glycogen synthase kinase 3β(GSK-3 β) and casein kinase 1 (CK1), which tags β-catenin and subsequently goes through UPS. Stabilization of β-catenin occurs with Wnt ligand binding to Frizzled receptors, allowing the degradation complex to be inactivated *via* low density lipoprotein receptor-related protein 5/6 (LDR5/6) and Disheveled. β-catenin accumulates and translocates into the nucleus where it couples with T cell factor/lymphoid enhancer factor (TCF/LEF) transcription factors to induce transcription of WNT target genes, Cyclin D-1 (CCND1), c-MYC, and Jun. β-catenin plays a crucial role in the self-renewal and differentiation of CSCs (278, 280, 281). The anomalous activation of Wnt/β-catenin is either through genetic alterations such as mutations in CTNNB1, the APC gene and AXIN genes, or through epigenetic modulation (282–284).

In breast and colorectal cancers, aberrant Wnt/β-catenin pathway activation is carried out by DNA methylation in the promoter region and silencing of multiple Wnt inhibitors such as Wnt inhibitory factor 1 (WIF-1), AXIN2, Secreted frizzled-related protein 1 (SFRP-1), and Dickkopf-related protein 1 (DKK1) (285–287).

Histone modifications are also implemented in the deregulation of the Wnt/β-catenin pathway in cancer. Decreased acetylation of H3K16 and increased H3K27 trimethylation, along with the recruitment of Sirtuin 1 (SirT1), enhancer of zeste homolog 2 (EZH2) and suppressor of zeste 12 protein homolog (Suz12) (components of polycomb repressor complex 2, PCR2) to the DKK1 promoter inhibits the expression of the DKK1 Wnt antagonist (288). Bivalent histone modifications, activating H3K4me3 and repressing H3K27me3 histone marks at its locus, are implemented in colorectal cancer by regulating Disheveled-binding antagonist of β-catenin 3 (DACT3). In turn, DACT3 expression in colorectal cancer lines is decreased, with overexpression of Wnt/β-catenin and CSC induction (289).

#### Hedgehog-Signaling Pathway

As an important mediator of embryogenesis and tissue homeostasis, Hedgehog (Hh) signaling has been shown to maintain SC and regulate the proliferation of progenitor cells (290). In the absence of the Sonic Hedgehog ligand (SHH), inhibition of Smoothened (Smo) protein by Patched receptor (PTCH-1) activates kinesin family member 7 (Kif7) and suppressor of fused homolog (SUFU), resulting in sequestration of Gli proteins which function as transcription factors. Moreover, upon binding of SHH to PTCH-1, Smo activates Hh signaling by releasing Gli protein back to the nucleus and exerting transcription of target genes (291). The implication of Hh mutation-induced signaling alterations in SCs has been well-documented in medulloblastoma and basal cell carcinoma. The upregulation of SHH within hair follicles or the interfollicular dermis in basal cell carcinoma was shown to contribute to tumorigenesis (292, 293). Moreover, granule neuron progenitors, identified as the medulloblastoma cell of origin, were seen to possess high levels of Hh signaling activity (294). In addition to genetic mutations, epigenetic factors were also seen to impact Hh-signaling. The chromatin remodeling protein SNF5 was seen to directly alter Hh signaling by interacting with Gli, resulting in the downregulation of PTCH-1 and resultant loss of the Hh inactivation feedback loop (295). Furthermore, it has been shown that hypomethylation of the SHH promoter allowed NF-κB to bind to the promoter, resulting in higher expression of SHH in gastric and breast cancer cells (296). Overexpression of SHH has been linked to CSC renewal and cancer aggressiveness (297).

#### Notch Signaling Pathway

The Notch signaling pathway is a highly-conserved cell signaling system that plays a major role in the regulation of embryonic development. It also regulates cellular proliferation and differentiation amongst a vast range of cell types and stages of cell maturation. Its cell-dependent signaling consists of the binding of ligands Jagged-1/-2 or Delta1-4, which triggers cleavage of the Notch intracellular domain (NICD) by γ-secretase, followed by release into the cytoplasm (298). This allows for modulation of SC differentiation and self-renewal, crucial for the survival and maintenance of neural stem cells (NSCs) (299).

In multiple myeloma, epigenetic histone acetylation causes overexpression of Jagged-2 ligand (300). Histone acetylation is governed by histone deacetylase (HDAC), and the recruitment of HDACs to the promoter regions is usually carried out by nuclear co-repressor silencing mediator of retinoic acid and thyroid hormone receptor (SMRT). In multiple myeloma, decreased levels of SMRT reduces HDAC recruitment to the Jagged-2 promoter, which in turn increases histone acetylation and increases Notch ligand transcription, ultimately resulting in overactivation of the Notch signaling pathway.

Serine-threonine kinase receptor-associated protein (STRAP) promotes tumorigenesis and stemness by stabilizing intracellular fragment of NOTCH3 (ICN3), notable in colorectal cancer. STRAP inhibits histone methylation of H3k27 at the HES5 and HES1 promoters, leading to gene overactivation and inducing treatment resistance (301).

### Cancer Stem Cells: Drivers of Therapy Resistance

CSCs and EMT-induced heterogeneity convey resistance to chemotherapeutic agents such as cisplatin, gemcitabine, and 5-fluorouracil (5-FU) (302, 303). Pancreatic cell lines exhibiting resistance to gemcitabine expressed high ZEB1 and low E-cadherin, thus acquiring great migratory ability (304). Tumor cell response to therapy may largely be due to epigenetic modulations.

With the enhanced expression of drug efflux transporters such as multidrug resistance-associated protein 1 (MRP1) and ATP-binding cassette sub-family G member 2 (ABCG2), drug resistance is increasingly observed (305–307). Transporter expression is regulated by various pathways and mechanisms, and deregulation results in protein enrichment and drug efflux. Notch signaling upregulates MRP1 expression and is responsible for CSC drug resistance (308, 309). The modification of histones (decreased HDAC1, and increased H3K4 tri-methylation, H3S10 phosphorylation, and histone H3 acetylation) leads to upregulation of ABCG2 expression. Along with decreased H3K9 tri-methylation, this allows for chromatin remodeling protein Brahma-related gene 1 (Brg1) and RNA polymerase II to gain access to the promotor, ultimately activating ABCG2 transcription (310). As a result of aberrant epigenetic modifications, physiologic SCs are susceptible to deregulation that facilitates tumor progression and invasion. Epigenetic regulation of signaling pathways is thus a potential target for anti-CSC therapy.

Heterogeneity is omnipresent in mammalian cells, and fundamental with regard to CSCs (311). The complicated picture of CSC heterogeneity involves dynamic cell populations capable of undergoing spontaneous state transitions; spontaneous switches from non-SCs to stem-like cells was observed in a study of breast cancer cells where plasticity was regulated by ZEB1 (312, 313). CSC heterogeneity and plasticity in various cancers varies from patient to patient, but phenotypically distinct CSC markers may be identified depending on the tumor genotype (311, 314). Non-CSCs and CSCs in breast cancer exhibited a dynamic equilibrium that was maintained by cytokine-mediated crosstalk among marked populations. This suggests that cancers have reversible phenotypic plasticity and do not solely depend on genetic variation (315, 316). Colorectal cancer studies have provided compelling evidence demonstrating CSC plasticity and tumor progression. The Wnt target gene LGR5 acts as a functional CRC marker. Anti-cancer drug therapy resulted in the conversion of LGR5+ into LGR5- cells, while in the absence of the drug, LGR5- reverted back to LGR5+ (317). CSCs have been shown to overcome DNA damage induced by radio/chemotherapy. Furthermore, they acquire resistance through overactivation of DNA repair mechanisms such as the expression of excision nucleotide repair protein ERCC1 and overexpression of cell cycle checkpoints (318, 319). CSCs have also been shown to inactivate cell cycle gene expression as well as apoptosis-inducing genes such as p53 and c-MYC, creating so-called “undruggable phenotypes” (320). The activation of autophagy pathways after exposure to cytotoxic agents induces apoptosis; unfortunately, this mechanism is a double-edged sword and has been shown to instead enable CSCs and a heterogeneous subpopulation of cancer cells to tolerate the cytotoxic agents and TME-induced stress. These cells enter a state of dormancy and degrade key transcription factors (i.e. p53) to prolong cellular survival until TME conditions become favorable for growth and proliferation (321, 322).

### Hypoxia: The Master Regulator of Cellular Heterogeneity

Hypoxia develops as a result of malignant cell overgrowth relative to their angiogenic requirements. To elicit cellular viability and progression, tumor-associated cells increase secretion of hypoxia-inducible factors (HIFs), mainly HIF-1-α and HIF-2-α, which in turn regulates angiogenesis in a chaotic manner (323). Under normal conditions, HIF-1-α is kept in check by hydroxylase enzymes which are dependent upon intracellular oxygen concentration. They are ubiquitinated and degraded after tumor suppressor protein von Hippel-Lindau complex formation. In hypoxic conditions, hydroxylation is diminished resulting in the overexpression of HIFs (324). The resultant chaotic blood vessel formation leads to irregular oxygen delivery and decrease in oxygen perfusion leads to necrosis (325). Drug distribution varies greatly between well-perfused and hypoxic areas, and effective cancer therapy requires efficient tumoral penetration; this patchy blood vessel distribution unfortunately results in tumor cell survival (326). HIF-1-α has been shown to upregulate various transcription factors (i.e. ZEB1/2, TWIST and SNAIL) that reduce E-cadherin expression, which results in EMT (327). Additionally, HIF-1-α activates focal adhesion kinase and steroid receptor coactivator (FAK-Src) which also decreases E-cadherin, promoting the EMT and VEGF-dependent angiogenesis and drug resistance by formation of SC-like phenotypic variants resistant to chemotherapy (328, 329). Intra-tumoral hypoxia induces a harsh environment that is crucial for cellular heterogeneity. The reprogramming of cellular phenotypes and metabolism drives adaptation and enhances signaling pathways leading to treatment resistance. As such, it is associated with poor prognosis (330). In a recent study, different patients with the same cancer type were shown to possess different inter- and intra-tumoral phenotypes. Very low oxygen concentrations correlates with an increase in mutational load in individual cells, and in varying the degree of hypoxia in each patient, alternations in tumor suppressors and oncogenes as such Myc, PTEN, and TP53 was elicited (331). HIF-1-α was shown to be a key player in the regulation of multiple metabolic pathways (i.e. amino acid metabolism, lipid metabolism, glycogenesis, and the TCA cycle) which ensures cancer cell sustenance and resistance to treatment (332, 333). A robust understanding of the HIF-1-α expression pathomechanism is required before we may implement effective therapeutic regimens.

### Metabolism of the TME

#### Lactate Metabolism

Metabolic reprogramming occurs when it is necessary to increase cellular proliferation under hypoxic conditions. It has been shown that cancer cells increase metabolism of reactive oxygen species, lactate, lipids, amino acids, glutamine and glucose (334). Under normoxic conditions, normal cells general energy through oxidative phosphorylation, while cancer cells employ lactate metabolism and glycolysis. It was previously shown that tumor cell production of lactate occurs via: 1) glycolysis using lactate dehydrogenase (LDH), which converts pyruvate into lactate, bypassing the TCA cycle; and 2) glutaminolysis which forms various metabolites, including lactate and pyruvate, allowing the cell to hijack the TCA cycle and utilize glucose-derived metabolism for better efficiency (335, 336). As glucose concentration within the TME is scanty, numerous tumor types (i.e. lung adenocarcinoma, pancreatic adenocarcinoma, and more) have shown very high expression of lactate dehydrogenase which is known to induce the EMT (337). Furthermore, a high-lactate TME has been shown to reprogram TME cells. The high lactate environment prevents the proliferation of cytotoxic and effector T cells while promoting immunosuppressive Tregs (338); it has also been shown to induce M2 polarization of TAMs, subsequently leading to recruitment of other Tregs to enhance the protection of the TME. High lactate content promotes survival of hypoxic cells by inducing angiogenesis (339). Glutaminolysis provides a source of nitrogen, carbon, and energy to fuel the stromal and cancer cells (340). A recent study pointed out the importance of glutamine metabolism, demonstrating that breast cancer cells used the pyruvate metabolite within the TME to effect ECM remodeling, inducing cancer cell stemness and resistance to anti-tumor agents (341, 342). The role of lactate in treatment resistance has been well documented. After irradiation of non-small cell lung cancer (NSCLC), mice xenografts showed resistance within six weeks (343). The importance of lactate as a key molecule in resistance mechanisms has been further elucidated in epidermal growth factor receptor (EGFR) and tyrosine kinase- (TK) targeted therapies. These treatment modalities prompted cancer cell lactate production, which directed TME cells to produce hepatocyte growth factor (HGF), ultimately resulting in EMT and resistance (344). Lactate metabolism was shown to increase DNA repair mechanisms by exploiting DNA-dependent protein kinases (DNA-PK), rendering cells resistant to cisplatin and doxorubicin (345).

#### Lipid Metabolism

Most neoplasms of organs and tissues are associated with adipocytes. The high rate of cellular proliferation demands abundant fuel *via* a process called lipid metabolic reprogramming. Lipid metabolism reprogramming has been correlated with resistance to conventional chemotherapeutic agents. Lipid and lipoproteins result from either catabolic processes or *de novo* synthesis (346). *De novo* fatty acid synthesis – lipogenesis – is controlled by the upregulation of lipogenic enzymes, and several crucial lipogenic enzymes such as fatty acid synthase (FASN), acetyl co-A carboxylase, stearoyl-CoA-desaturase-1 (SCD-1) and ATP citrate lyase are highly expressed in most neoplastic cells (347). High lipogenic enzyme concentration is correlated to invasion and worse prognosis (348). Upregulation of the prominent enzyme FASN is complex. It may be mediated by various growth factors, such as EGFR, HER2, steroid hormone receptors-androgen receptors, estrogen receptors and progesterone receptors; release is induced by the harsh conditions of the TME, or may result from post-translational miRNA modifications (349). Another key contributor in lipogenesis is SCD1, which is upregulated by growth factors (i.e. EGFR, PDGF, TGF-β) within the TME, and has been associated with treatment resistance and worse prognosis (350, 351). Various studies showed that inhibiting FASN and SCD1 action in lipogenesis led to tumor regression and improved responsiveness to prior therapeutic resistance (352). Another means by which various cancers may derive energy metabolites is *via* lipolysis. Overexpression of fatty acid-binding protein-4 (FABP-4), which induces lipolysis, has been shown to contribute to rapid tumor growth, metastasis in ovarian cancer and resistance to carboplatin (353). CAAs provide cancer cells with exogenous free fatty acids through cancer cell phenotypic expression of surface fatty acid translocase (CD36) through the fatty acid beta-oxidation (FAO) pathway (162). The CD36+ subpopulation have been shown to be more aggressive and resistant to treatment (354). In another study of radiotherapy-resistant breast cancer cells and breast cancer SCs, carnitine-palmitoyl-transferase-1a-and-2 (CPT1a/2), a known contributor to the FAO pathway was shown to be highly expressed. When CPT was knocked out by genetic editing techniques, this rendered previously-resistant cells sensitive to radiotherapy (355). The TME demonstrates atypical lipid metabolism for cell membrane formation and production of energy (356). Lipid metabolism has been linked to cancer growth, recurrence (357) and CD8 T cell exhaustion *via* the upregulation of programmed-cell death protein-1 (PD-1) (358) resulting in post-chemotherapy evasion of immune surveillance. The derangements of lipid metabolism are especially crucial for CSCs as the high ectopic metabolism of lipids has been linked to CSC formation, self-renewal and pluripotency (359). In obese breast cancer patients, sustained elevation of IL-6 and FGF-2 was observed. Obese mouse breast cancer xenografts also showed resistance to anti-VEGF therapy; the pathomechanism is hypothesized to be the constant release of proinflammatory cytokines by adipocytes. IL-6 and FGF-2 blockade restored sensitivity of cancer cells to anti-VEGF therapy (360). The association between drug resistance and lipid metabolism reprogramming has been well-documented in the literature (**Table 2**).

**Table 2:** Pharmaceutical agents or medical interventions for which a TME-regulated resistance mechanism has been described.

Cancer type	Pharmaceutical agent or intervention	Mechanism of action	Resistance mechanism	Reference
LIPOGENIC				
Breast cancer (*in vitro*)	Tamoxifen	Inhibition of Estrogen Receptor	Alterations within the cholesterol pathway were prominent in all resistant cell lines	(361)
NSCLC (*in vivo*)	Gefitinib	Inhibits EGFR	SCD-1 upregulation induced resistance to gefitinib by promoting the EGFR-signalling pathway. Inhibition of SCD-1 rendered the cells responsive to gefitinib	(362)
AML (*in vitro*)	Mitoxantrone	Inhibitor of Topoisomerase II	Cellular visualization showed an increase in lipid droplet accumulation. Genetic analysis from sensitive and resistant cell lines showed that resistant cell lines had significantly higher mitochondrial activity and oxidative phosphorylation (OXPHOS) indicating an increase in fatty acid synthesis. OXPHOS inhibitors reversed cellular resistance	(363)
HNSCC (*in vitro)*	Radiation therapy	Double-strand DNA breaks	Glucose uptake was shown to be high in cells, and decrease in mitochondrial OXPHOS was apparent. Resistance was achieved through increased expression of fatty acid synthase (FAS). Combination treatment with FAS inhibitors induced cytotoxicity to resistant cells	(364)
LIPOLYTIC				
AML (*in vivo*)	Cytarabine	Nucleoside analog	An increase in fatty acid beta-oxidation (FAO) was observed, with high mitochondrial OXPHOS and CD36 expression. Targeting the FAO-OXPHOS-CD36 axis rendered the cells sensitive to conventional therapy.	(365)
Multiple cancer models	Anti-angiogenic therapy	Inhibitor of VEGF-R	VEGF inhibitors induced lipid metabolic reprogramming by increasing free fatty acid levels through increased CPT-1 expression, thus causing resistance. Inhibition of CPT-1 re-sensitizes previously resistant cells to anti-VEGF.	(366)
Breast cancer *(in vitro)*	Paclitaxel	Anti-microtubule	Activation of the JAK/STAT3 pathway confers resistance to breast cancer and breast cancer stem cell lines. Inhibition of JAK/STAT3 led to inhibition of CPT-1b and abolished CSC self-renewal capabilities.	(367)
Melanoma *(in vitro/vivo)*	Inhibitors of BRAF/MEK	Selectively inhibits mitogen activated protein kinases	To acquire resistance, cells switch from the glycolytic pathway to oxidative respiration by peroxisomal FAO. Knockdown of peroxisome key enzymes (acyl-CoA oxidase-1) or treatment with peroxisomal FAO inhibitor resulted in a durable anti-tumor response.	(368)

NSCLC, non-small cell lung cancer; AML, acute myeloid leukemia; HNSCC, squamous cell carcinoma of the head and neck; SDT-1, Stearoyl-CoA-desaturase-1; CPT-1, carnitine-palmitoyl-transferase-1a; CSC, cancer stem cell.

#### Reactive Oxygen Species Metabolism

Reactive oxygen species (ROS) elevation is closely related to cancer severity due to its influence over tumor immunity, tumorigenesis, and cellular reprogramming (369). Under hypoxic conditions, HIFs are activated by local mitochondrial ROS, and are therefore implicated in angiogenesis (370). ROS are produced by various cells within the TME, inducing activation of the KRAS pathway and promoting tumorigenesis (369). ROS were shown to play a critical role in the activation of TAMs, MDSCs and CAFs, enhancing their immunosuppressive roles (123, 371). Therapy resistance remains the most challenging barrier in cancer treatment. The pioneer in cancer metabolism, Otto Warburg first observed that cancer cells rely on glycolysis rather than oxidative phosphorylation, and this shift from oxidative to reductive metabolism is now termed the “Warburg effect” (336). Although glycolysis is considered an inefficient mode of energy production, ATP can be provided to cells at a faster and safer rate compared to the TCA cycle, which induces more stress *via* ROS formation (372). Upregulation of the glycolytic pathway aids cellular proliferation by shunting metabolites (glycine, serine, alanine) and nucleotides to the pentose phosphate pathway (PPP) (373). Transketolase, a key enzyme in the PPP was shown to increase pyrimidine synthesis and induce resistance to gemcitabine (374). ROS are the consequence of aerobic metabolism, and the major sources are peroxisomes, mitochondria and NADPH oxidase. Under physiologic conditions, redox homeostasis with low levels of ROS is maintained through fluctuations in generation and elimination processes, as an elevation in ROS is detrimental and leads to cell death. In cancer cells, metabolic derangement and oncogenic signaling induces high production of ROS (375, 376). Mitochondria are susceptible to ROS-induced oxidative damage, which usually results in elevation of NADPH oxidase expression. This in turn favors glycolysis and decreases the intrinsic production of ROS (377, 378).

- ROS-mediated maintenance of glycolysis: Pyruvate kinase (PK) is a rate limiting enzyme of the glycolytic pathway, and appears in two isoforms termed M1 and M2. PKM1 has high kinase activity and is present in physiologic conditions whereas PKM2 exhibits low pyruvate kinase activity which prevents its entrance in the TCA cycle; PKM2 is highly expressed in cancer cells (379). PKM2 was shown to activate HIF-1-α-related genes (i.e. LDHA, SLC2A1) after hypoxia-induced anti-angiogenic therapy (380). Furthermore, the low activity of PKM2 induces glutathione reduction in order to counter the effects of ROS accumulation after ROS-producing therapies (381). Another important glycolytic enzyme termed the “housekeeping gene”, glyceraldehyde-3-phosphate-dehydrogenase (GAPDH) is upregulated in tumors and is associated with cancer aggressiveness (382). GAPDH maintains glycolysis by redirecting metabolites to the PPP in order to induce an increase in NADPH. A study showed that changes in glucose concentration enhanced NADPH oxidase-dependent ROS production, leading to resistance to doxorubicin (383). Upregulation of glycolysis has been shown to enhance DNA repair mechanisms after chemo- or radiation therapy (384). Inhibition of the glycolytic pathway re-sensitized cells to previously resistant drugs (385, 386).

- ROS-mediated activation of oncogenic signals:

Adenosine monophosphate protein kinase (AMPK), a key element of tumor suppression that prevents the Warburg effect was shown to possess tumor-supporting actions, inducing metabolic variations to sustain the ROS-damaged cellular mechanism (387). Apart from its angiogenic functions, HIF-1-α induces the expression of glycolysis-associated genes (i.e. GLUT1/3, hexokinases, and PKM2) to maintain glycolysis and inhibit the TCA cycle (388). The “guardian of the genome”, p53, functions to maintain genome integrity after DNA-induced damage. It has also been shown that p53 acts as a negative regulator of the Warburg effect (389). ROS-induced damage impairs p53 activity and prevents apoptosis (390). Furthermore, ROS metabolism has been linked to treatment-associated metabolic disturbances (391). Chemo- and radiation therapy induce cancer cell death *via* ROS production, and ROS production has been shown to induce the activation of oncogenic signaling pathways (NF-κB and PI3/Akt) which ensures cell survival against the ROS onslaught (392). Well-documented drug efflux mechanisms induce MDR (ABC transporters, i.e. P-glycoprotein) (393, 394). Eventually, TME cells acclimate to ROS and become resistant to ROS-eliciting drugs by producing antioxidants or increasing efflux of cytotoxic agents (395, 396).

#### Acidic TME

As a result of hypoxia and high lactate, TME niches are acidic. This harsh environment induces oncogene activation, and cellular metabolism shifts to adapt (397). Compared to normal cells, cancer cells possess a high intracellular pH which promotes proliferation and inhibits apoptosis, and maintains a low extracellular pH in a “reversed pH gradient” (398, 399). The acidic niche acts synergistically with the effects of lactate by inducing TAM M2 polarization, and inhibiting the cytotoxicity of infiltrating T cells (400). These effects support cellular development (401) and regulate immune surveillance. The acidic niche has been shown to induce invasiveness and the EMT in melanoma (402), neuroblastoma (399, 403) and breast carcinoma cells (404). The pH gradient between intra- and extracellular spaces forms a physical barrier to weak-base chemotherapy, preventing proper drug uptake and distribution through physiological resistance or “ion trapping phenomenon”. Ionization of weak-base agents within the acidic extracellular environment prevents them from traversing this barrier (405, 406).

In contrast, weak acids exhibit high intracellular permeability. For example, paclitaxel, a non-ionizable agent, was not impeded by this physiologic barrier, showing how the ion trapping hypothesis may be relevant in future treatment modalities (407). This has prompted researchers to alkalinize appropriate modalities or treatment combinations prior to administration. Low pH brought about by hypoxia and low perfusion was shown to induce epigenetic modifications, mainly in p53, preventing apoptosis and increasing activity of P-glycoprotein in order to induce MDR (408, 409). It has been previously reported that the acidic TME was involved in cellular protection against irradiation (410). In an investigation of radio- and/or chemo-resistance, a study showed that the acidic niche functions to induce cellular dormancy by arresting the cell cycle at G2/M phase (411). Finally, another mechanism of treatment resistance depends on the genomic instability generated by acidic milieu, which induces phenotypic variations that lead to cellular stemness (412).

#### Immune Micro-Environment Variability Between Primary and Secondary Tumors

As previously described, the vast cellular repertoire within the TME contributes to immune suppression. Secretion of soluble factors within the TME prevents active immune surveillance from entering the tumor; these are known as “cold tumors” – low-immune infiltrates that enhance proliferation, migration and invasion. 90% of cancer-related deaths occur in the metastatic stage because of the inefficient localization of micro-metastatic niches and therapeutic failure (413). A study utilized deep learning was conducted to detect micro-metastatic niches, and this innovative technique enabled metastatic analysis of mice with metastatic lung, pancreatic and breast cancers that may potentially be treatable. Antibody-targeted treatment applied to visible metastatic nodules was also distributed to the micro-niches in close vicinity. This approach provides the means to identify micro-niches distributed throughout the body for the purposes of improving treatment efficacy (414). The TME-induced heterogeneity was more evident in another study that showed discrepancies in the cellular and immune repertoire within primary and metastatic lesions. This suggests yet another therapeutic resistance mechanism (415). In addition to immune suppression, the microenvironmental repertoire of immune cells has been implicated in treatment resistance. As stated earlier, a large portion of the TME consists of bone marrow-derived myeloid cells which are modulated by both physical and biochemical signals that cause them to differentiate. Myeloid cells include TAMs, TANs and MDSCs which were all shown to induce chemo- and radio-therapeutic resistance through a variety of mechanisms.

TAMs are the predominant myeloid cell type within the TME, and their differentiation into the M2 phenotype is an important factor in treatment resistance. An influx of TAMs is observed after the initiation of therapy (416). TAMs were shown to be key players in chemotherapy resistance, producing various inflammatory mediators such as TNF-α, MMP, cathepsin and TGF-β. They are also commonly described as EMT transducers, degrading and synthesizing denser ECM, which ultimately leads to treatment resistance (416, 417). Another resistance mechanism is *via* TAM production of signaling factors such as FGF-2, IL-8 and VEGF for angiogenesis (418, 419). TAMs were shown to sustain an elevated level STAT3 activation, which has been associated with chemo- and radiotherapy resistance. Elevated STAT3 inhibits apoptosis *via* upregulation of anti-apoptotic proteins bcl-2 and IAP (420). Similarly, TAM overexpression of EGFs such as milk fat globule EGF-8 (MFG-E8) was shown to induce overactivation of the Sonic Hedgehog and STAT3 pathways in CSCs, resulting in treatment resistance to cisplatin (418, 421).

As previously described, microenvironmental recruitment of TANs results in a high likelihood of N2 polarization. In addition to their immune modulatory effects, they have been implicated in ECM remodeling and the EMT through the secretion of proteins such as HGF, MMP and oncostatin-M (422, 423). Similar to TAMs, TANs also induced angiogenesis, promoting treatment resistance *via* secretion of Bv8, MMP9 and VEGF (424). Additionally, HCC xenografts showed an increase in TAN activity and an increase in the expression of chemokines such as CCL2 and CCL17, which serve to attract Tregs and macrophages to the TME, thus inducing resistance to sorafenib. Pharmacologic inhibition of the PI3K-AKT pathway was shown to decrease levels of CCL2 and CCL17 chemokines and re-sensitize cells to sorafenib (425).

MDSCs are a major determinant of immunogenicity. Through the production of TGF-β, MDSCs induce polarization of TAMs and TANs into their respective tumor-supporting subtypes (150). IL-10 oversecretion by MDSCs was shown to inhibit anti-tumor activity by preventing macrophage activation and DC maturation (426, 427). The receptor tyrosine kinase inhibitor sunitinib malate was shown to not only reduce the suppressive function of MDSCs, but also decrease the expression of Fox-p3, TGF-β and IL-10, inducing a significant increase in anti-tumor activity (428). An *in vivo* study of multiple myeloma xenografts showed neutrophil accumulation in the bone marrow in the course of disease. The accumulation of MDSCs and Tregs is thought to be a result of cancer expression of stem-cell factor ckit ligand (429). The resistance of multiple myeloma to melphalan and doxorubicin is due to the immunosuppressive actions of MDSCs, mediated by soluble factors, and it is hypothesized that targeting the MDSCs would enhance chemotherapeutic efficacy in this cancer (430). Furthermore, reprogramming the TME immune repertoire induces a better anti-tumor activity and more robust response to chemotherapy (431).

As described above, CAFs are one of the key mediators of ECM stiffness and myeloid cell differentiation. CAFs differentiate from various stromal cells of the TME. Despite advancements in oncological treatments, the prognosis of solid tumors such as HCC and pancreatic ductal adenocarcinoma remains poor. Firstly, CAF-dependent secretions promote ECM rigidity, which prevents effective drug penetration. Secondly, CAF-derived miRNAs previously shown to induce immune suppression were also shown to induce treatment resistance. Ovarian cancer cells showed downregulation of programmed cell death 4 (PDCD-4) in the presence of CAF-derived overexpression of miR-182. MiRNA-182 alterations of PDCD-4 expression rendered the cancer cells resistant to chemotherapy (432). Cisplatin-based therapy was administered to patients with esophageal cancer; high levels of CAF-derived miR-27a were subsequently observed. MiR-27a-dependent transformation of fibroblasts into CAFs resulted in optimal production of TGF-β, and is thought to be the mechanism of therapy resistance. Inhibition of TGF-β subsequently re-sensitized the cells to cisplatin (433). Thirdly, CAF-derived exosomal release promotes cancer aggressiveness and therapy resistance. This occurs when the EMT is induced by modulating Wnt-PCP autocrine signaling, which is further involved in cellular polarity *via* JNK and ROCK pathways (434). CAF-derived exosomes were shown to induce therapy resistance in breast cancer cells *via* juxtacrine and paracrine signaling of STAT-1 and NOTCH-3 pathways (435). Additionally, STAT-1 and NOTCH-3 have been associated with the maintenance of cancer cell stemness, which has been further associated with oxaliplatin and 5-FU resistance in colorectal cancer (436). Within the TME, CAFs were shown to hyperactivate the Wnt/β pathway, which in turn induces the expression of ABC and P-glycoprotein (437, 438). Overactivation of the Wnt pathway not only results in chemo- and radiotherapy resistance, it has also been shown to reduce intracellular ROS through the overexpression of COX-2 and aldehyde dehydrogenase (439, 440).

Although the molecular interplay between treatment resistance and immune suppression is not yet fully elucidated, these novel resistance mechanisms induced by TAMs, TANs, MDSCs and CAFs may present the future for targeted therapy.

#### Mechanical TME

The importance of ECM remodeling as a result of mechanical changes has been well established. Multiple studies demonstrate that tension accumulated in the TME induces an increase in metabolism for: 1) rapid proliferation (441); 2) mobility and structural changes that regulate invasion (442), and 3) immune evasion, acquired epigenetic modification by miRNA, and stress-induced signaling that induces resistance to therapy, which collectively constitute the most threatening aspect of cancer cell dormancy (443, 444). It has been shown that physical signals can alter cellular behavior beyond the traits of CSCs (445). The TME – with dense interstitial matrix, abnormal blood and lymphatic vessels, and increased stromal pressure – is physically distinct from normal tissue (446). Physical signals of the TME include increased matrix stiffness, solid stress and interstitial fluid pressure. Operating in tandem, these physical factors contribute to treatment resistance (445, 447).

### Increased ECM Stiffness

ECM composition determines its rigidity. As described previously, the ECM provides crucial biochemical and structural support for the TME and is comprises of two components: 1) polysaccharides, which assemble into proteoglycans; this forms a gel-like structure in which fibrillar proteins embed; and 2) fibrillar proteins (fibronectins, laminins, collagen and elastin) which function as ligands for cell adhesion molecules (448). ECM proteins are produced by mesenchymal cells and the constant restructuring of the ECM is modulated by hormones, growth factors, cytokines and extracorporeal factors which influence homeostasis, repair mechanisms and morphology (449). A key aspect of ECM remodeling focuses on mesenchymal cell (i.e. fibroblast)-mediated proteolysis and re-synthesis, which is dependent on the activity of MMPs and LOX, respectively. During re-synthesis, CAFs express high levels of LOX which cross-links collagen and elastin thus increasing the rigidity of the ECM. Another stiffening mechanism, previously described, is the constant inflammatory state of the TME that induces fibroblastic transformation into myelofibroblasts. The level of desmoplastic reconstruction is positively associated with treatment resistance and worse prognosis (450). A meta-analysis showed that the level of ECM stiffness was positively correlated with cancer cell genomic instability. Three hypotheses were proposed by the authors: 1) stiffness induces DNA damage during cellular migration; 2) tumor invasion of a densely-packed environment results in the selection of more aggressive phenotypes; and 3) stiffness enhances proliferation (451). It was hypothesized that a shift from the physiologic basement membrane to a collagen-rich, dense and rigid ECM is a key factor in therapy resistance (452).

Cellular stiffness within the TME is exerted *via* transmembrane proteins, mainly integrins. Integrins exhibit dual function when exposed to stress:

1. as messengers that interact with intracellular-signaling pathways (kinases such as FAK/Src, MAPK, ROCK, JNK) and anti-apoptotic oncogenes (e.g. the YAP/TAZ/HIPPO pathway) delivering mechanical signals from surrounding cells to the transcription apparatus of the nucleus (453, 454). This activates integrin and kinase overexpression, inducing phenotypic variation and EMT (455);
2. physically connects to actin components of the cytoskeleton *via* linker proteins (e.g. vinculin, α-actinin and talin), signaling molecules (FAK, Src), and adapter proteins (Paxillin, senescent cell-antigen-like containing domain 1, PINCH-1) to modify cytoskeletal contractile forces and thereby inducing the EMT (456, 457).

The modification of the nuclear envelope, with regard to cancer cell progression has been described in a number of studies (458). The nuclear envelope mainly consists of the nuclear pore complex and lamins, which link the nuclear and cellular cytoskeletons, and both were shown to be greatly modulated by cancer cells (458). A mechano-sensor, the nuclear envelope converts and transmits signals to the nucleus, thus dictating nuclear deformability (459). This parameter in turn regulates cellular plasticity and invasion of dense tissue (460). Nucleus-cytoskeleton interactions influence nuclear stiffness, which impacts chromatin rearrangement, transcription of previously repressed genes, and change in cellular polarity. These interactions are shown to support resistance to therapy and facilitate the metastatic process (461).

Multiple cancer models have established how ECM rigidity influences chemotherapeutic resistance and cancer proliferation. Breast cancer is resistant to sorafenib, a result that is positively correlated with collagen concentration and degree of stiffness. Furthermore, triple negative breast cancer (TNBC) cells exhibit resistance as a result of overexpression of β1-integrin dependent activation of the JNK pathway (462). Moreover, another study cultured TNBC cells in varying degrees of ECM stiffness before exposing them to doxorubicin, and doxorubicin efficacy is seen to be negatively correlated with ECM stiffness. Nuclear translocation of YAP in those cells appears to be the primary driver of the EMT (463). Another well-studied entity is HCC which presents with extensive fibrosis. HCC resistance to paclitaxel, cisplatin, 5-FU and sorafenib is shown to be positively correlated with ECM stiffness (462, 464). High stiffness-ECM was seen to induce HCC dormancy, with expression of SC markers such as CD133, CXCR4 and NANOG (465). Furthermore, ECM stiffness has been shown to mediate HCC stemness and resistance to oxaliplatin. The resistance mechanism appears to depend on integrin expression in response to ECM-mediated stiffness, which in turn upregulates phosphorylation of the Akt/mTOR pathway that is crucial for self-renewal (466). These investigations show how ECM stiffness mediates treatment resistance, utilizing a cascade of signals that originate from cell-cell and cell-ECM connections, and are a potential target to mitigate treatment resistance.

### Growth-Induced Solid Stress

Solid stress arises from mechanical (shear, compressive, and tensile) forces exerted by the elastic and solid components of the TME. Rapid cellular proliferation, infiltration, and ECM deposition leads to the ready accumulation of solid stress in the TME, and becomes a significant barrier to effective drug delivery. Furthermore, solid stress collapses vessels and initiates cellular dormancy; after conventional treatments deplete sensitive cancer cells, these dormant cells with stemness are reawakened and nourished by the blood vessels (467–469). Solid stress induces hypoxic failure of chemo- and radiotherapy delivery, while hypoxia-mediated HIF-1-α has the capacity to induce EMT and encourage the development of cells with SC phenotypes (470). Moreover, a study showed that solid stress induced the upregulation of ECM adhesion molecules and the formation of “leader cells”. These are capable of coordinating cellular migration, resulting in cellular invasion and metastasis (471). A boost in leader cell phenotypes has been observed following exposure to conventional treatments (472). Demonstrating high transcriptional plasticity, leader cells have been shown to possess CSC-like properties with resistance to chemo- and radiotherapy (473).

#### Interstitial Fluid Pressure

High interstitial fluid pressure (IFP) is also dependent on ECM stiffness. This is caused by hypoxia-induced angiogenesis and impaired vessel function, which has been associated with resistance to targeted therapy, chemo-, and radiation therapy (474, 475). IFP increase in tumors has not been fully explained but it is thought to occur after leakage in defective vessels, followed by high protein deposition, contributing to ECM rigidity (476). This was particularly apparent in pancreatic ductal adenocarcinoma, where it was observed that high hyaluronan content collapsed vessels and decreased cytotoxic drug distribution. This mechanical resistance was reversed after enzymatic breakdown of the stroma (477).

The mechanical changes within tumors render otherwise effective chemo- and radiotherapeutic approaches ineffective. In addition to the physical barrier to therapy, the stiffened ECM was shown to induce the EMT and the development of cellular dormancy. As was described in these three mechanisms of resistance, early combination therapies targeting the cancer type and aspects of the physical blockade could increase efficacy and prevent the development of therapy-resistant variants.

#### TME Innervation

It has been well established that cancer invasion can occur into or around nerve routes *via* perineural invasion (PNI) (478); this has been associated with pain and poor prognosis (479). It has been shown that PNI induces the release of factors necessary for tumor growth (480), and consequently, the cells of the TME were seen to induce an adrenergic neuronal cell phenotype which supports metastasis in pulmonary (481), ovarian (482) and pancreatic cancers (483, 484). Prostate cancer studies have shown that cancer cells express neurotrophic growth factor (NGF), which attracts nerve fibers toward the TME to promote tumor invasion and metastasis (485). Additionally, denervation has been shown to suppress tumorigenesis, further denoting the importance of innervation (486). Although apparently significant, neurotransmitter concentration in serum was not sufficiently elevated, and it is now thought that perhaps this increased concentration is diverted towards the TME. Additionally, it has been shown that astrocytomas were able to resist treatment modalities by forming a tight microenvironment covered by a microtubular network resistant to radiotherapy (487), and expressing phenotypic changes within the TME that induced stemness, which resulted in chemotherapeutic resistance (488).

**Figure 2:**
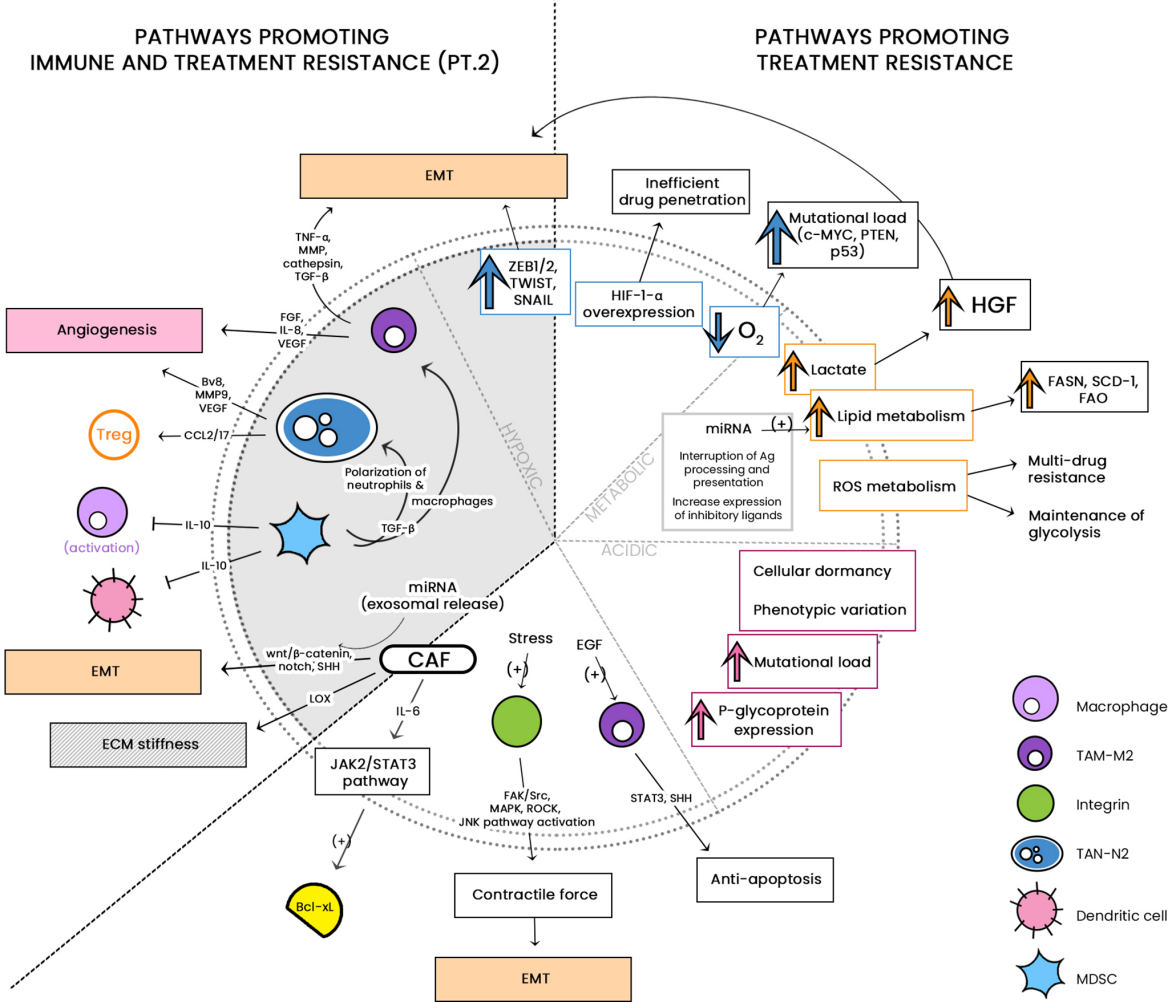
Alterations in the tumor microenvironment (TME) induce modifications in metabolic pathways and mechanical stress. These alterations have been shown to induce drug resistance by amplifying cell-to-cell and cell-to-ECM crosstalk, activating protective pathways and inducing phenotypic variations, in addition to biochemical signaling and resistance to apoptosis. Moreover, soluble factors released by tumor-supporting immune cells have been shown to induce both immune and drug resistance *via* induction of epithelial-to-mesenchymal transition (EMT), extracellular matrix (ECM) stiffness and angiogenesis. TGF-ß, Transforming growth factor- ß; LOX, lysyl oxidase; TNF- α, tumor necrosis factor alpha; ROS, reactive oxygen species; ECM, extracellular matrix; HGF, hepatocyte growth factor; HIF-1-α, hypoxia inducible factor-1-alpha; VEGF, vascular endothelial growth factor; CCL, C-C motif chemokine ligand; MAPK, mitogen activated protein kinase; EMT, epithelial-to-mesenchymal transition; IL, interleukin; JNK, c-Jun-N-terminal kinase; JAK/STAT, Janus kinase/Signal transducer and activator of transcription; ROCK, Rho-associated protein kinases; FAK/Src, Focal adhesion kinase/src family kinase; FASN, fatty acid synthase; Bv8, prokineticin 1; FGF, fibroblast growth factor; FAO, fatty acid oxidation; SCD-1, stearoyl-CoA-desaturase-1; MMP, matrix metalloproteinase.

See **Figure 2** for a diagrammatic summary of the major pathways that promote treatment resistance, and immune and treatment resistance.

## Conclusion

Within the complex microenvironment of the TME, immune progenitors are encouraged to differentiate into regulatory T cells, M2 macrophages and MDSCs, amongst others, rather than fulfilling their tumor-suppressive roles as fully mature immune cells. The interaction between cellular components and soluble factors of the TME efficiently nurtures immune evasion and suppression, drug resistance, and promotes malignancy. Cellular crosstalk *via* both paracrine and juxtracrine signaling coordinates key elements that define cancer stemness, extracellular matrix remodeling, and the recruitment of non-malignant tumor-supporting cells. In addition to immune resistance, therapy resistance within the TME is achieved through various physical and biochemical factors that induce the EMT and modulate epigenetic changes that result in the formation of CSCs. In this review, it is evident that landmark research has elucidated these dysfunctional immune components with increasing clarity. Many of these components are now targets of promising drug therapies currently undergoing investigation, and these ground-breaking new discoveries continue to pave the way for new treatment modalities in the fight against cancer.

## Author Contributions

Conceptualisation, KK; Investigation and Resources, KK, DH, JC, CS; Writing - Original Draft Preparation, KK, DH, JC, CS; Writing - Review and Editing, KK, JC, MK; Visualisation, KK, JC, MK; Graphics, JC, KK; Supervision, AM, MK; and Project Administration, AM, MK. All authors contributed to the article and approved the submitted version.

## Conflict of Interest

The authors declare that the research was conducted in the absence of any commercial or financial relationships that could be construed as a potential conflict of interest.
